# O3-2 Evaluation of implementation and impact of a national mass media campaign to promote active lifestyles in Portugal: ‘Follow the Whistle: Physical Activity is Calling You'

**DOI:** 10.1093/eurpub/ckac094.018

**Published:** 2022-08-29

**Authors:** Marlene Nunes Silva, Cristina Godinho, Marta Salavisa, Catarina Santos Silva, Rute Santos, Romeu Mendes, Katherine Owen, Pedro Teixeira, Adrian Bauman

**Affiliations:** 1 National Program for Physical Activity Promotion-PNPAF, Directorate-General for Health, Lisbon, Portugal; 2 CIPER, Faculdade de Motricidade Humana, Universidade de Lisboa, Lisbon, Portugal; 3 CIS-IUL, Universidade Católica Portuguesa, Lisbon, Portugal; 4 Research Centre in Physical Activity, Health and Leisure, Faculty of Sport, University of Porto, Porto, Portugal; 5 EPIUnit, Instituto de Saúde Pública, Universidade do Porto, Porto, Portugal; 6 School of Public Health, Sydney University, Sydney, Australia

**Keywords:** Mass media national campaign, Process and impact evaluation, COM-B model, Social Marketing

## Abstract

**Background:**

Portugal has one of the highest rates of physical inactivity in Europe. To raise the profile of physical activity (PA) among adults, the Portuguese National Physical Activity Promotion Program of the Directorate-General of Health developed and implemented a national mass media campaign in 2019 named ?Follow the Whistle?. The campaign was based on social marketing principles and behavior change theory, namely the COM-B model, and was developed through formative research. We aim to describe the implementation process and short-term impact evaluation data.

**Methods:**

‘Follow the Whistle' was launched in June 2019 and continued for 4 weeks (TV, Radio, Online and Cinema, newspapers, outdoors). Pre (n = 878, 57%women) and post (n = 1319, 58%women) independent adult population samples were used to assess campaign impact. Participants were recruited through the mailing list of a Portuguese mutual insurance company, a network from across Portugal. The online questionnaire comprised socio-demographic factors, campaign awareness and recall, and psychosocial and behavioral measures linked to the four targets of the COM-B model, including items to assess perceived capability for physical activity(C), perceived opportunity for practice/ ease of integration of PA in daily living(O), motivation for PA practice(M) and behavior(B) assessed with IPAQ and the Activity Choice Index.

**Results:**

Pre-and post-campaign samples were broadly demographically similar. Campaign recall increased significantly from 1% to 24% in prompted tagline recall (1/3 at post-campaign correctly recalled specific campaign images). All the targeted COM-B indicators changed in the expected direction, suggesting increases in perceived capability to engage in PA, perceived daily opportunities for being physically active, and in motivation (considering PA as fun, important, and compatible with other important things in life) (all p > 0.05). Results from PA behavior measures revealed increases only for vigorous activities (p > 0.001).

**Conclusions:**

The GAPPA recommends the creation of active societies starting with best-practice community-wide communications campaigns. Although one focus of the ?Follow-the whistle? (i.e. promotion of active transport and incidental everyday forms of physical activity) was not entirely achieved (i.e. changes only in vigorous activities), the initial campaign objectives of increases in awareness, and influencing intermediate outcomes were achieved. Further campaign waves are planned for future years.

